# Antioxidant and Antihyperlipidemic Effects of* Campomanesia adamantium* O. Berg Root

**DOI:** 10.1155/2016/7910340

**Published:** 2016-07-14

**Authors:** Priscilla Pereira de Toledo Espindola, Paola dos Santos da Rocha, Carlos Alexandre Carollo, Wanderlei Onofre Schmitz, Zefa Valdivina Pereira, Maria do Carmo Vieira, Edson Lucas dos Santos, Kely de Picoli Souza

**Affiliations:** ^1^Federal University of Grande Dourados, Rodovia Dourados-Itahum, Km 12, 79804-970 Dourados, MS, Brazil; ^2^Federal University of Mato Grosso do Sul, Cidade Universitária, s/n, 79070-900 Campo Grande, MS, Brazil

## Abstract

*Campomanesia adamantium* O. Berg, popularly known as guavira, has been used in Brazilian traditional medicine for reduction of serum lipid. The present study was carried out to investigate the antioxidant and antihyperlipidemic effects of* Campomanesia adamantium* root aqueous extract (ExCA). Phenolic compounds were quantified in the ExCA and gallic and ellagic acids were identified by HPLC. ExCA showed efficiency in 2,2-diphenyl-1-picrylhydrazyl free radical scavenging, with IC_50_ similar to butylhydroxytoluene control, and protected the erythrocytes against lipid peroxidation induced by 2,2′-azobis(2-methylpropionamidine) dihydrochloride, reducing generated malondialdehyde. Hyperlipidemic Wistar rats treated daily by gavage during eight weeks with ExCA (200 mg/kg of body weight) showed reduced serum level of total cholesterol and triglycerides, similar to normolipidemic rats and hyperlipidemic rats treated with simvastatin (30 mg/kg of body weight) and ciprofibrate (2 mg/kg of body weight). Moreover, the treatment with ExCA also decreased malondialdehyde serum level in the hyperlipidemic rats. The body weight and organ mass were unmodified by ExCA in hyperlipidemic rats, except an increase of liver mass; however, the hepatic enzymes, alanine aminotransferase and aspartate aminotransferase, were unchanged. Together, these results confirm the potential value of* Campomanesia adamantium* root for lowering lipid peroxidation and lipid serum level, improving risk factors for cardiometabolic diseases development.

## 1. Introduction

Dyslipidemia is characterized by higher serum level of total cholesterol and triglycerides, accompanied by reduction of high-density lipoprotein (HDL). In 2008, more than 17.3 million people died from cardiovascular diseases [1], such as atherosclerosis, which can lead to stroke and myocardial infarction [2].

Concomitant with increased serum lipid level, decreasing of antioxidant capacity of the organism has been observed, which contributes to endothelial dysfunction present in atherosclerosis and connects this alteration with modified metabolic state [3]. Reactive species formation occurs continuously in the body especially in consequence of oxidative metabolic process for energy generation. In lower concentration, these molecules have physiological function in cellular signalization and proliferation. However, in higher concentration it reactive species lead oxidative damage to protein, lipid, and nucleic acid, affecting key cellular structures [4].

The body has endogenous mechanisms, enzymatic and nonenzymatic, for neutralizing the excess of reactive species and decreasing possible cell damages [5]. The excess of oxidants agents which are not neutralized defines the oxidative stress present in dyslipidemia, obesity, and atherosclerosis [6].

Medical plants, rich in vitamins and secondary metabolites, have been source of inhibitors of the endogenous synthesis of cholesterol, as well as natural antioxidants [7, 8]. In Brazil, the fruit of* Campomanesia adamantium* O. Berg (Myrtaceae) is used for nutrition and, in the traditional medicine, the leaves and root are used for treatment of diabetes and dyslipidemia. Similar effects have been described for other species of the genus such as hypolipidemic and antiplatelet effects [7, 9], antiulcerogenic effects [10], reducing body weight [11], and antidiabetic effects [12]. Additionally, phytochemical studies have showed phenol and flavonoids in the leaves of* Campomanesia adamantium*, which has been described as molecules with high potential antioxidant [13].

In this context, this study was carried out to investigate the antioxidant and antihyperlipidemic effects of the root aqueous extract of* Campomanesia adamantium* in rats with high fructose diet-induced hyperlipidemia (HFD).

## 2. Materials and Methods

### 2.1. Botanical Material and Obtaining Extract

*Campomanesia adamantium* O. Berg roots were collected in Dourados, MS, under coordinates S 22°02′47.9′′ W 055°08′14.3′′. They were sanitized, dried in an oven with air circulation at 45°C, and ground in a Willy-type knife mill. A voucher specimen was deposited in the herbarium DDMS/UFGD number 4108.

The extract was prepared by repeat extractions of the pulverized material using accelerated solvent extractor (ASE® 150-Dionex). The samples were placed in a cell of 100 mL and extracted with distilled water at a temperature of 125°C in static two cycles of 5 min each time, with 80% of the volume of washing and 60-second purge. The extracts were combined in an aqueous medium and then lyophilized to obtain the dry extract, yield 6%.

### 2.2. High Performance Liquid Chromatography Coupled with Diode Array Detector (HPLC-DAD)

ExCA chemical profile was determined by HPLC-DAD (Shimadzu SPD-M20A, Japan) using a reversed phase column C-18 (250 mm × 4.6 mm, 5.0 *μ*m, Shimadzu, Japan) mobile phase water (A) and acetonitrile (B), both with 1% acetic acid. The elution gradient was 5% of eluent B over 3 min and 3–60% of eluent B over 3–27 min and another 6 min to return the initial condition and restabilize the column. The flow rate was 1.0 mL/min and the injected volume was 20 *μ*L. Compounds were quantified at 270 nm in triplicate analysis. The quantification was performed using a regression curve of five points, 0.5–50 *μ*g/mL for gallic acid (Sigma-Aldrich, USA) and 2.5–100 *μ*g/mL for ellagic acid (Sigma-Aldrich, USA). Analyzed tracks were linear (detector response/concentration). Peak areas were correlated with the concentration according to the calibration curve and the correlation coefficient (*r*) > 0.9998 for both compounds.

### 2.3. 2,2-Diphenyl-1-Picrylhydrazyl (DPPH) Free Radical Scavenging

The activity of 2,2-diphenyl-1-picrylhydrazyl (DPPH) free radical scavenging was available according to D. Gupta and R. K. Gupta [14] method. Briefly, 200 *μ*L of extract (0.1–2000 *μ*L) was mixed with 1.8 mL of DPPH 0.11 mM in 80% ethanol solution. The mixture was homogenized and incubated at room temperature in the dark for 30 min. The reading was performed in a spectrophotometer at 517 nm. Ascorbic acid and butylhydroxytoluene (BHT) were used as reference antioxidants. The tests were performed in triplicate in three independent experiments. The percentage of inhibition was calculated relative to the control (ascorbic acid and BHT) using the following equation:

(1) inhibition  activity %=1−absorbance  of  sampleabsorbance  of  control×100.

### 2.4. Protection against 2,2′-Azobis(2-Methylpropionamidine) dihydrochloride (AAPH) Induced Hemolysis

The antioxidant activity of the extract was determined by erythrocytes protection capacity against hemolysis induced by peroxyl radicals generated by thermal decomposition of the hydrochloride 2,2′-azobis(2-methylpropionamidine) dihydrochloride (AAPH), following the methodology described by Campos et al. [15]. For this, peripheral blood of healthy donors was collected into tubes containing anticoagulant (approved by the ethics committee of the Federal University of Grande Dourados under protocol number 123/12). Then, the blood sample was centrifuged at 1500 rpm for 10 min. After centrifugation, the plasma cell and the upper layer containing leukocytes were removed, preserving only the lower layer comprising red cells. The erythrocytes were washed three times with the addition of approximately 4 mL of saline and centrifuged at 2000 rpm for 10 min. After washing, a suspension of 10% erythrocytes was prepared. The* Campomanesia adamantium* extract was solubilized in 0.9% NaCl at concentrations of 50, 75, 100, and 125 *μ*g/mL. The same procedure was carried out with ascorbic acid (an antioxidant standard).

In 250 *μ*L of 10% erythrocyte sample, 250 *μ*L aliquots were added at their respective concentrations analysis (50, 75, 100, and 125 *μ*g/mL); this mixture was incubated at 37°C for 30 min, and then 500 *μ*L of AAPH (0.406 g of AAPH diluted with 15 mL of 0.9% NaCl) was added. The material remained incubated at 37°C under gentle mixing for up to 180 min. Aliquots of 200 *μ*L samples were removed and diluted in 1800 *μ*L of 0.9% NaCl for analysis in a spectrophotometer at 540 nm at 60 min intervals. The percentage hemolysis was determined by measuring the absorbance of the supernatant (*A*) and compared to the total hemolysis (*B*), using the following equation:

(2) $$\begin{array}{c} \text{hemolysis}\;\left(\;\%\;\right)=\frac{A}{B}\times 100. \end{array}$$

### 2.5. Lipid Peroxidation of Erythrocytes Induced by 2,2′-Azobis(2-Methylpropionamidine) (AAPH) dihydrochloride

We collected 5 mL of healthy blood donor for the preparation of the erythrocytes solution (H) 20%. The malondialdehyde (MDA), a product of lipid peroxidation of the erythrocyte, was performed as described by Campos et al. [15]. Samples of H (2.5%), H+AAPH, H+AAPH+ascorbic acid, and H+AAPH+ExCA were incubated for 180 min at 37°C. Concentrations available were 50, 75, 100, 125, 250, and 500 *μ*g/mL of ascorbic acid and ExCA was added to an aliquot of 0.5 mL of each sample and 0.5 mL of 20% trichloroacetic acid. An aliquot of 0.5 mL was removed from this mixture and added to tubes containing 1 mL of the reagent thiobarbituric acid (TBA) 10 nM incubated at 94°C for 45 min. After this period, the samples were kept at room temperature for 15 min, followed by addition of 3 mL of butanol, followed by agitation and centrifugation at 3000 rpm. The supernatant absorbance was read in a spectrophotometer at 532 nm, and three independent assays were performed in triplicate.

### 2.6. High-Fructose Diet-Induced Hyperlipidemia

High-fructose diet was prepared by mixing 330 g of commercial rodent chow (Labina) and 660 g of fructose and presented caloric value of 377 ± 4 kcal/100 g of chow. Commercial rodent chow (Labina) with caloric value of 332 ± 1 kcal/100 g of chow was used as control.

### 2.7. Experimental Design

#### 2.7.1. Animals

All procedures with animals were performed in accordance with the ethical principles of animal experimentation adopted by the National Council of Animal Experimentation Control (CONCEA) and were approved by the Ethics Committee on Animal Use (CEUA) of the Federal University of Grande Dourados (UFGD) under the protocol number 025/2012.

Wistar rats with about 60 days weighing between 140 and 150 g of body weight (BW) were pretreated with ad libitum diet rich in fructose for 16 weeks to induce hyperlipidemia, which was confirmed by the serum levels of total cholesterol, 79.0 ± 2.9 to 128.0 ± 14.5 mg/dL, and triglyceride levels of 137.3 ± 23.0 to 243.3 ± 32.4 mg/dL. After this assessment, the animals continue receiving the diet rich in fructose and were randomly divided into four experimental groups (*n* = 8 per group) as follows: HFD (high-fructose diet + 300 *μ*L of water), HFD-S (HFD + 30 mg of simvastatin by kg of BW), HFD-C (HFD + 2 mg ciprofibrate by kg of BW), and HFD-ExCA (HFD + 200 mg of* Campomanesia adamantium* root aqueous extract by kg of BW). For in vivo treatment, the lyophilized extract, ciprofibrate, and simvastatin were dissolved in distilled water daily before use and administered by gavage.

Animals fed with standard rodent commercial chow and 300 *μ*L of water by gavage (*n* = 8 per group) formed the CT group and they were considered normolipidemic.

#### 2.7.2. Organs and Tissues Available and Biochemical Analysis

After euthanasia the liver, heart, lung, kidney, spleen, soleus, and extensor digitorum longus (EDL) muscle were isolated and weighed. The collected blood was centrifuged at 3000 rpm for 10 min and the serum was used to measure total cholesterol, HDL cholesterol, triglycerides, and aminotransferases (AST and ALT) with support of equipment Integra 400 Plus (Roche*™*).

#### 2.7.3. Dosage of Malondialdehyde (MDA)

The concentration of MDA as index of lipid peroxidation was determined by incubating 200 *μ*L of serum from the animal of the groups HFD and HFD-ExCA for 45 min at 93°C with 1 mL of 10 nmol TBA. After this period, the samples were kept at room temperature for 15 min and added to 3 mL of butanol with subsequent agitation and centrifugation for 5 min at 3000 rpm. The supernatant absorbance was performed at 532 nm [15].

#### 2.7.4. Liver Lipids

Quantification of total lipids in the liver was performed in lyophilized sample subjected to extraction with chloroform and methanol (2 : 1) according to Association of Official Analytical Chemists, 1970.

### 2.8. Statistical Analysis

Data are shown as the mean ± standard error of the mean and were submitted to one-way analysis of variance (ANOVA) followed by Student-Newman-Keuls posttest. The results were considered significant when *P* < 0.05.

## 3. Results

### 3.1. Chemical Profile

**Figure 1:**
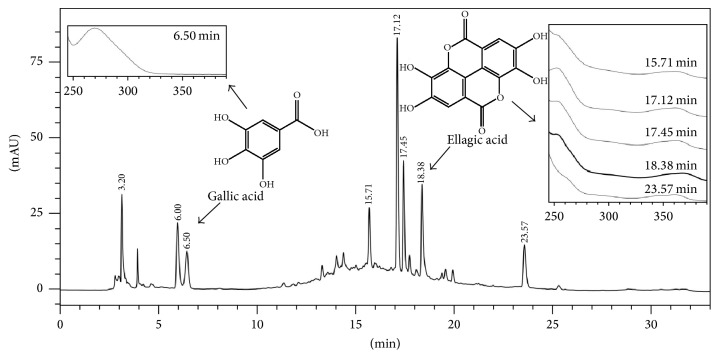
Identification of compounds in the aqueous extract of* Campomanesia adamantium* roots by HPLC-DAD (270 nm).

The chemical profile of the extract showed the presence of gallic acid in 6.5 min and ellagic acid in 18.38 min and other metabolites containing the same chromophore of the ellagic acid (Figure 1). The concentration of gallic acid and ellagic acid was 2.83 ± 0.01 and 15.48 ± 0.50 *μ*g/mg of ExCA, respectively.

### 3.2. Antioxidant Activity

Considering the presence of antioxidants, gallic acid, and ellagic acid in the extract, we investigated the DPPH free radical scavenging activity in order to determine the in vitro antioxidant activity. From the various concentrations tested, the half-maximal inhibitory concentration (IC_50_) of DPPH free radical scavenging and the maximum activity of ExCA were calculated, which are shown in Table 1. IC_50_ of ExCA was similar to BHT but was about 15 times higher than that of ascorbic acid.

**Figure 2:**
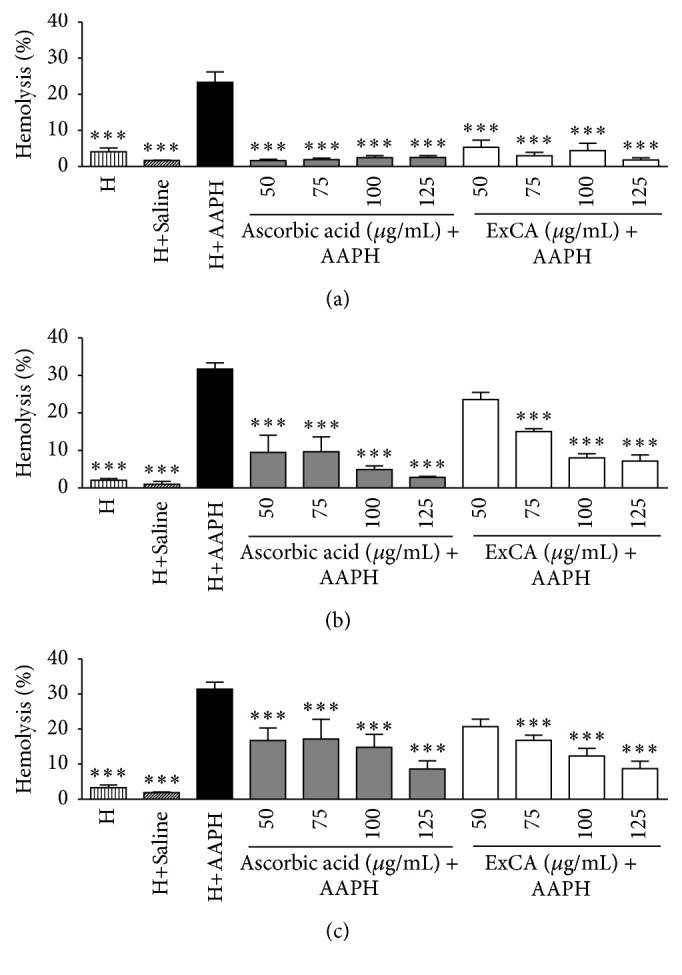
Hemolysis assessment at (a) 60, (b) 120, and (c) 180 min after addition of AAPH in 2.5% erythrocytes (H) incubated with different concentrations (50–125 *μ*g/mL) of ascorbic acid and* Campomanesia adamantium* root aqueous extract (ExCA). ^*∗∗∗*^ *P* < 0.001 versus H+AAPH.

After checking the DPPH free radical capture capability, we investigated the antioxidant activity of ExCA in in vitro cellular model. The ExCA protected the human erythrocyte of the hemolysis induced by AAPH until 180 min at all concentrations evaluated similarly to ascorbic acid, except in the concentration of 50 mg/mL (Figure 2).

**Figure 3:**
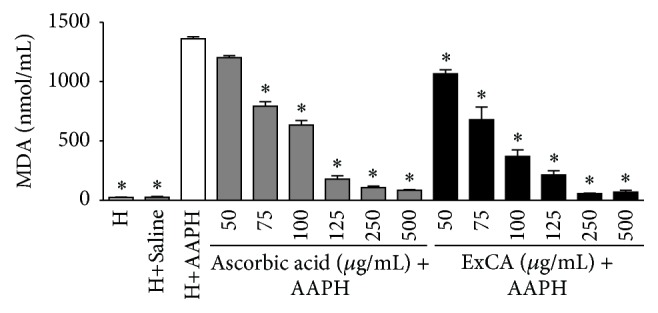
Malondialdehyde (MDA) content at 180 min after addition of the AAPH hemolysis inducer in 2.5% erythrocytes (H) incubated with different concentrations (50–500 *μ*g/mL) of ascorbic acid and* Campomanesia adamantium* root aqueous extract (ExCA). ^*∗*^ *P* < 0.001 versus H+AAPH.

The ability of the ExCA to protect against AAPH-induced lipid peroxidation of human erythrocytes was evaluated through the dosage of MDA. ExCA provided concentration-dependent reduction in the generation of MDA similar to ascorbic acid (Figure 3).

### 3.3. Hypolipidemic Activity

**Figure 4:**
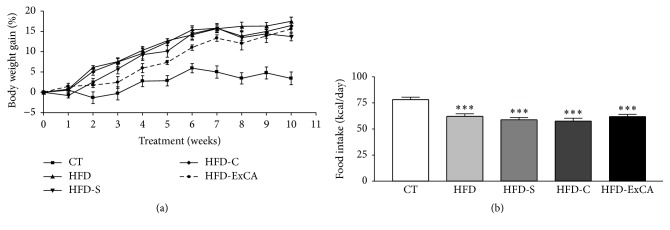
(a) Body weight gain and (b) daily caloric intake of normolipidemic (CT) and hyperlipidemic rats treated with water (HFD), simvastatin (30 mg/kg of BW, HFD-S), ciprofibrate (2 mg/kg of BW, HFD-C), and* Campomanesia adamantium* root aqueous extracts (200 mg/kg of BW, HFD-ExCA) during eight weeks by gavage. *n* = 8 in each group. ^*∗∗∗*^ *P* < 0.001 versus CT group.

During the eight weeks of treatment, the evolution of body weight was similar between all the animals fed with HFD (Figure 4(a)) and higher than CT group. However, a reduction in caloric intake of animals in HFD groups was observed, compared to the CT group (Figure 4(b)).

The assessment of organ weights reveals that HFD-ExCA and HFD-C groups showed recovery of the liver mass compared to CT group and higher than HFD group (Table 2). Additionally, no differences were observed in serum levels of liver injury markers (AST and ALT) and other organs investigated (Table 2).

**Figure 5:**
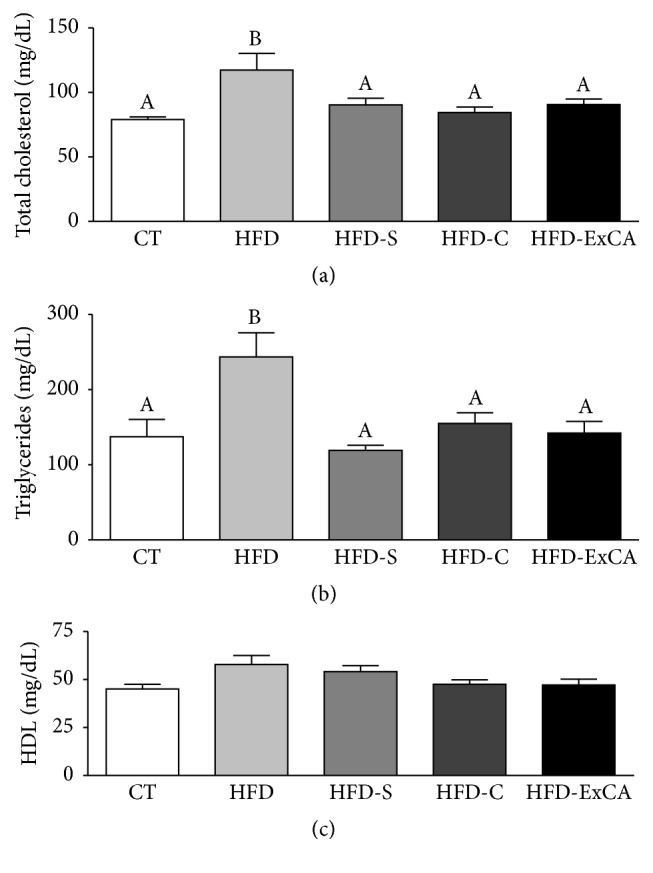
(a) Total cholesterol, (b) triglycerides, and (c) HDL cholesterol serum levels of normolipidemic (CT) and hyperlipidemic rats treated with water (HFD), simvastatin (30 mg/kg of BW, HFD-S), ciprofibrate (2 mg/kg of BW, HFD-C), and* Campomanesia adamantium* root aqueous extracts (200 mg/kg of BW, HFD-ExCA) during eight weeks by gavage. *n* = 8 in each group. Different superscript letters indicate significant differences at *P* < 0.05 between groups.

Confirming the antioxidant activity of ExCA, we investigated the lipid lowering in vivo activity of the extract. Animals treated with ExCA show decreased serum levels of total cholesterol and triglycerides, when compared with hyperlipidemic animals of the HFD group, similar to those presented by the animals treated with simvastatin (HFD-S), ciprofibrate (HFD-C), and normolipidemic animals (CT) (Figures 5(a) and 5(b)). There was no change in HDL serum levels among the groups investigated (Figure 5(c)).

**Figure 6:**
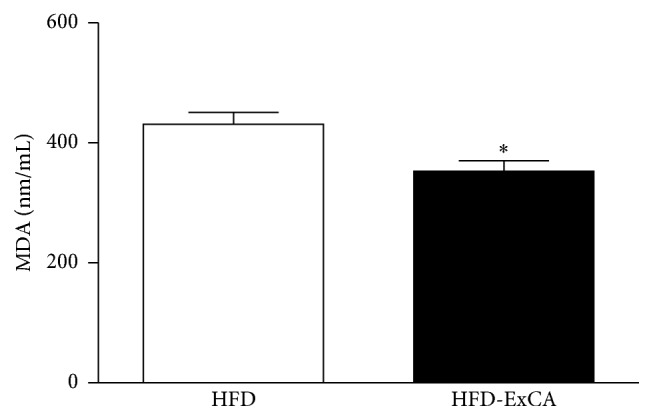
Malondialdehyde (MDA) serum level in hyperlipidemic rats treated with water (300 *μ*L, HFD) and* Campomanesia adamantium* root aqueous extract (200 mg/kg of BW, HFD-ExCA) during eight weeks by gavage. *n* = 8 in each group. ^*∗*^ *P* < 0.05 versus HFD group.

Considering the chemical constituents detected in ExCA and its antioxidant activity as well as reduction of serum lipids, we investigated the serum levels of malondialdehyde (MDA) in vivo. The hyperlipidemic animals treated with ExCA show decreased MDA serum levels compared to controls of the HFD group (Figure 6).

## 4. Discussion

Our results show by first time that the* Campomanesia adamantium* root aqueous extract reduces serum levels of total cholesterol and triglycerides similar to the reference drugs used for treatment of dyslipidemia and possesses antioxidant properties. These activities probably are due to its chemical constituents, partially the ellagic and gallic acids derivatives present in the ExCA, and other unidentified phenolic compounds including flavonoids.

Phenolic compounds are among the major classes of secondary metabolites of plants and several studies have demonstrated their capacity for radical free scavenging [16, 17].

The ellagic and gallic acids derivatives, which present in ExCA, have a direct action in the capture of free radicals [18] and, in addition, are capable of stimulating the activity of the antioxidant enzymes, superoxide dismutase (SOD), catalase (CAT), and glutathione peroxidase (GPx) [19, 20], contributing to the antioxidant actions observed. The action of the ExCA in the capture of free radicals was demonstrated in this study and its possible action on antioxidant enzymes may be evaluated in future studies.

The activity of free radical scavenging contributes to lower generation of malondialdehyde (MDA). This is a toxic product resulting from the degradation of the cell membrane by lipid peroxidation which leads to release of unsaturated fatty acids and phospholipids disintegration, causing cell ruptures and mutations [21]. The ExCA reduced the generation of MDA in erythrocytes induced with AAPH, which probably contributes to the conservation of the cell structure. This effect demonstrated in vitro was confirmed in vivo, since reduction of MDA in the serum of hyperlipidemic animals treated with ExCA was observed. Amin and Nagy [22] correlated phenolic compounds present in the extracts of various plants with reduced peroxidation and production of MDA in rats treated with high-fat diet and protection against LDL oxidation.

The oxidation of the LDL decreases their interaction with specific membrane receptors and increases its permanence in the bloodstream, making oxidized LDL a proinflammatory marker [23, 24]. These structural and functional changes of LDL favor the development of atherosclerotic plaque in blood vessels, concomitant with the increase in lipid peroxidation products, such as malondialdehyde [25]. Antioxidants, as ExCA, can reduce oxidation of LDL and attenuate the atherosclerotic vascular lesion, lowering stroke occurrence under this condition [26]. Kannan and Quine [27] found that ellagic acid administered to rats in a model of hypertrophy and arrhythmias induced experimentally reduced hyperlipidemia and lipid peroxidation in these animals because of its ability to inhibit the activity of 3-hydroxy-3-methylglutaryl-CoA reductase. In addition, ellagic acid has anti-inflammatory activity [28, 29] and renal protective effect attributed to its antioxidant potential [19].

Medicinal plants, beyond antioxidant activity, have been widely used as lipid-lowering agents [22, 30] and several studies have confirmed this ability [9, 31, 32], as observed for ExCA, which was capable of reducing total serum cholesterol and triglycerides similar to drugs currently used for the treatment of hypercholesterolemia and hypertriglyceridemia, ciprofibrate and simvastatin, respectively. The concomitant reduction in cholesterol and triglycerides induced by ExCA is an advantage, since the hypertriglyceridemia rarely occurs as a single factor and is almost always accompanied by high levels of total cholesterol [33]. Thus, the search of new drugs that have broad effects and are less toxic and capable of reducing serum lipid and controlling oxidative stress associated with dyslipidemia is growing [20]. These effects were described by Usta et al. [34] who describe many benefits of ellagic acid that is present in many fruits and nuts with antioxidant power and lowering lipid. Although the liver mass of the animals treated with the ExCA has increased, the marker enzymes of liver damage (AST and ALT) were unchanged.

The mechanism involved in cholesterol reduction may be inhibition of the enzyme HMG-CoA reductase, as observed by Klafke et al., [7] for* Campomanesia xanthocarpa*. However, the reduction of triglyceride possibly involves increased lipolysis either by raising the catabolism of VLDL and chylomicrons or by the activity of lipoprotein lipase; mechanisms have not been described yet for* Campomanesia* gender.

Together, the results reveal the antioxidant effect* Campomanesia adamantium* root aqueous extract and its ability to lower lipid, making it promising to prevent or treat dyslipidemia and furthermore expand our knowledge of plants used in traditional medicine.

## Figures and Tables

**Table 1:** Antioxidant activity: IC_50_ and percentage of maximum activity of DPPH free radical scavenging of *Campomanesia adamantium* root aqueous extract (ExCA) compared to standard antioxidant, ascorbic acid, and BHT.

Sample	IC_50_ (*μ*g/mL)	*N*	Maximum activity	
			%	*μ*g/mL
Ascorbic acid	2.5 ± 0.6	(4)	98	25
BHT	36.1 ± 9.1	(4)	93	500
ExCA	37.3 ± 4.1	(4)	96	250

Values are expressed as mean ± SEM.

**Table 2:** Tissues, organs, and serum levels of AST and ALT of normolipidemic (CT) and hyperlipidemic Wistar rats treated with water (HFD), simvastatin (HFD-S), ciprofibrate (HFD-C), and *Campomanesia adamantium* root aqueous extract (HFD-ExCA).

Parameters	CT	HFD	HFD-S	HFD-C	HFD-ExCA
Soleus (g/100 g of BW)	0.03 ± 0.001	0.03 ± 0.003	0.03 ± 0.001	0.03 ± 0.003	0.02 ± 0.004
EDL (g/100 g of BW)	0.01 ± 0.002	0.01 ± 0.001	0.01 ± 0.001	0.01 ± 0.001	0.01 ± 0.002
Heart (g/100 g of BW)	0.32 ± 0.01	0.34 ± 0.01	0.33 ± 0.004	0.30 ± 0.04	0.31 ± 0.009
Kidney (g/100 g of BW)	0.60 ± 0.02	0.54 ± 0.01	0.61 ± 0.01	0.56 ± 0.08	0.59 ± 0.01
Liver (g/100 g of BW)	2.89 ± 0.03	2.71 ± 0.05	2.85 ± 0.06	3.08 ± 0.06^*∗∗∗*^	3.09 ± 0.07^*∗∗∗*^
AST (U/L)	174.0 ± 6.5	178.0 ± 14.5	219.5 ± 19.8	195.0 ± 15.9	154.1 ± 15.2
ALT (U/L)	53.2 ± 3.3	65.7 ± 7.0	64.8 ± 5.8	65.0 ± 4.1	71.9 ± 7.4

BW, body weight; EDL, extensor digitorum longus muscle; AST, aspartate aminotransferase; ALT, alanine aminotransferase. Mean ± SEM. ^*∗∗∗*^ *P* < 0.001 versus HFD group.
